# Beyond Screen Time: Parent–Adolescent Relationship as a Mediating Pathway Between Problematic Internet Use and Psychological Well-Being Among Malaysian Adolescents

**DOI:** 10.3390/bs16081341

**Published:** 2026-08-04

**Authors:** Ijeoma Miriam Okafor-Emeagha, Vimala Ramoo, Mary Joseph Marret, Lee Wan Ling

**Affiliations:** 1 Faculty of Nursing Science, Universiti Malaya, Kuala Lumpur 50603, Malaysia or ijay.emeagha@gmail.com (I.M.O.-E.);; 2 School of Nursing, Faculty of Health Sciences, Nursing and Education, Mahsa University, Bandar Saujana Putra, Jenjarom 42610, Selangor, Malaysia; 3 Department of Pediatrics, Faculty of Medicine, Universiti Malaya, Kuala Lumpur 50603, Malaysia

**Keywords:** adolescence, problematic internet use, psychological well-being, parental mediation

## Abstract

The construct of adolescent psychological well-being has long been a focus of both public discourse and scholarly inquiry. With the widespread adoption of the Internet, there have been growing concerns about adolescents’ exposure to escalating online risks. Parental mediation has been hypothesised to facilitate the guidance and regulation of adolescents’ Internet use. However, the specific mediating role of the parent–adolescent relationship in the association between problematic Internet use and psychological well-being has remained underexamined, a gap explored in the current study. Based on the theoretical underpinnings provided by Phase Three of Bronfenbrenner’s Bioecological Model of Human Development, a cross-sectional survey using validated psychometric instruments was conducted, involving 1598 students (mean age 16.0 years; 51% female) from secondary schools in Selangor, Malaysia. For data analysis, inferential statistics and partial least squares–structural equation modelling (PLS–SEM) were used to examine the complex structural relationships among the variables. Mean scores for problematic Internet use and psychological well-being indicated moderate severity levels. SEM results indicated the negative influence of problematic Internet use on psychological well-being (*β* = −0.104 *, *p* = 0.040) and parent–adolescent relationship (*β* = −0.241 **, *p* < 0.001) and a significant positive influence of parent–adolescent relationship on psychological well-being (*β* = 0.328 **, *p* < 0.001). Subsequent mediation analysis confirmed the partial mediating effect of parent–adolescent relationship (indirect association: *β* = −0.079) on the relationship between problematic Internet use and psychological well-being among Malaysian adolescents. This was consistent with Bronfenbrenner’s Bioecological Model of Human Development. Strengthening the parent–adolescent relationship could mitigate the adverse psychological effects of excessive Internet use. From a policy perspective, these findings supported the use of family-focused intervention as a strategy to address the adverse psychological effects of problematic Internet use among adolescents.

## 1. Introduction

Adolescence is a pivotal developmental stage characterised by rapid physical, cognitive, emotional, and social transformation. Within this developmental phase, adolescents attain greater independence, cultivate complex social identities, and establish behavioural patterns that have significant implications for their long-term well-being. In the 21st century, rapid advancements in digital technologies have reshaped adolescent experience. Internet use has become a central aspect of adolescents’ academic, social, and recreational lives, with global reports of more than 90% of adolescents in developed countries and an increasing proportion of adolescents in developing countries having access to the Internet daily (Hällgren & Björk, 2022). While the Internet offers substantial benefits, such as facilitating access to educational resources, supporting skill development, and fostering global communication, there are growing concerns regarding the potential of excessive or maladaptive use, including problematic Internet use (PIU). This condition is characterised by compulsive use of online platforms, loss of control over the usage patterns, and continued use despite negative consequences (Young, 1998). PIU has been linked to a range of adverse outcomes in diverse cultural contexts, including reduced academic performance, disrupted sleep, social withdrawal, and emotional distress (Aderinto, 2022). Global prevalence estimates range between 8% and 26%, depending on the definitions and measurement tools (Astarini & Yudiarso, 2020). In Southeast Asia, rapid digitalisation and high smartphone penetration have contributed to elevated Internet engagement, with Malaysia ranking among the top countries in the region for connectivity (Asian Development Bank & UN.Escap, 2018). Although this enhances digital inclusion, it increases the risk of PIU, thereby necessitating robust preventative interventions.

### 1.1. Adolescent Problematic Internet Use and Psychological Well-Being

Psychological well-being (PWB) is a multidimensional construct encompassing self-acceptance, autonomy, personal growth, purpose in life, environmental mastery, and positive relationships. The relationship between PIU and PWB among adolescents is complex and often bidirectional (Ryff, 1989). Studies have consistently reported the relationships of excessive Internet use with higher levels of depression, anxiety, and stress and diminished life satisfaction (Sanghvi & Rai, 2015). Conversely, adolescents seeking to alleviate psychological distress may utilise online platforms, such as social media and gaming, as coping strategies to obtain validation and escape offline challenges (Kostyrka-Allchorne et al., 2023).

### 1.2. Parental Mediation on Internet Use and Its Impact on Adolescent Psychological Well-Being

A positive parent–adolescent relationship (PAR) can mitigate the psychological effects of PIU. On the other hand, conflicts or distant relationships between adolescents and parents may heighten vulnerabilities, leading to greater engagement in maladaptive online behaviours (De Simone et al., 2025; O’Reilly & Mohan, 2023). PAR that fosters self-regulation, emotional resilience, and decision-making (Pontes & Macur, 2021; Lan et al., 2022) enhances adolescents’ capacity for association-based technology management. Parental involvement manifests in diverse modalities, including active mediation (through discussion of online content), restrictive mediation (by setting limits), and co-use through shared online activities (Livingstone & Helsper, 2008). Evidence suggests that active mediation is particularly associated with promoting healthier online behaviours and greater adolescent well-being (Giovanelli et al., 2025). However, the use of these different modalities may vary, depending on cultural norms and parenting styles, underscoring the importance of context-specific research.

### 1.3. Current Study

There are gaps in the literature on the direct influence of adolescents’ Internet use on PWB and the mediating role of PAR. There is a pressing need to fill these gaps, especially for countries in Southeast Asia, such as Malaysia, where cultural norms emphasise family cohesion (Faudzi et al., 2020). Understanding the interplays between these variables within an Asian society is necessary for comparison with existing studies from predominantly Western contexts that feature differing cultural norms and family dynamics (Ratten et al., 2023). This is crucial for formulating locally relevant and culturally sensitive strategies that balance the benefits of digital engagement with the need to safeguard adolescents’ well-being.

This study was underpinned by Bronfenbrenner’s Bioecological Model of Human Development, emphasising the dynamic interactions between individuals and their multiple environmental systems over time. According to this model, developmental outcomes are shaped by proximal processes, which are regular, sustained interactions with people, objects, and symbols in one’s immediate environment, and are associated with the characteristics of the individual and the surrounding context (Bronfenbrenner & Morris, 2007). In the context of this study, Internet use was conceptualised as a behavioural factor situated within the adolescent’s microsystem, in which frequent engagement with digital devices and online platforms is a significant part of daily interactions. PWB is a developmental outcome that may be adversely affected by PIU. PAR is a key microsystem factor that functions as either a protective or a risk factor, depending on the quality of the relationship.

**Figure 1:**
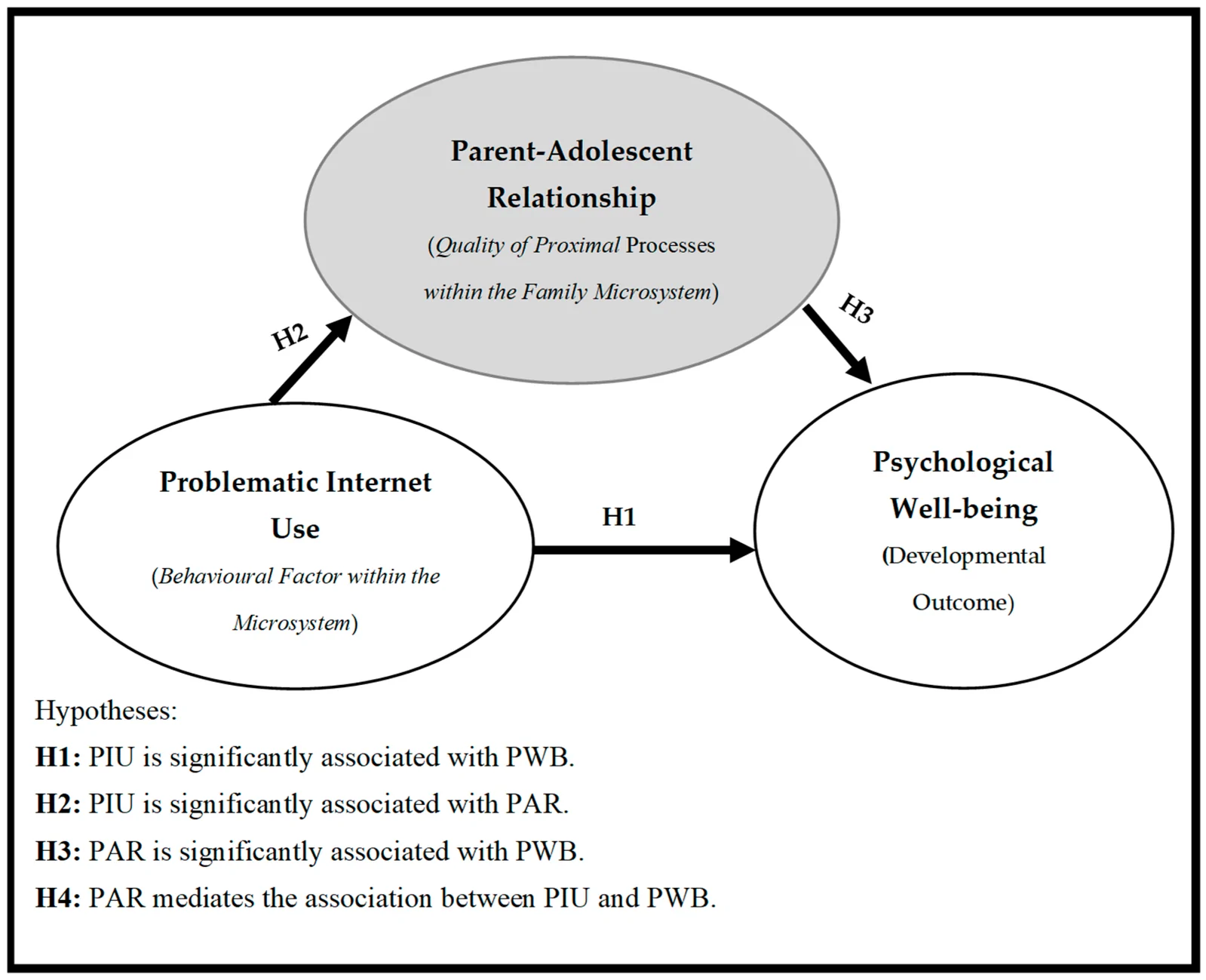
Conceptual Framework for the Study.

The framework posited that a strong parental relationship, characterised by care, trust, and open communication, can buffer the detrimental influence of PIU on PWB. Conversely, weaker parental relationship may exacerbate these associations, leading to poorer mental health outcomes. Guided by this theoretical lens, the model (Figure 1) illustrated the hypothesised direct relationship between PIU and PWB, as well as the mediating role of PAR in this association.

With that, this study aimed to assess the levels of PIU, PWB, and PAR; examine the relationships between PIU and PWB, PAR and PWB, and between PIU and PAR; and determine whether PAR mediates the relationship between PIU and PWB. The study’s findings would significantly contribute to the growing body of literature on the psychosocial impacts of Internet use during adolescence, thereby yielding practical implications for the development of school-based interventions, parental guidance programmes, and national digital well-being strategies. Furthermore, the study’s incorporation of a theoretically grounded model and its focus on a culturally specific context offered globally relevant and locally applicable insights, enabling a comprehensive understanding of the phenomenon in question.

## 2. Materials and Methods

### 2.1. Study Design

An analytical cross-sectional survey was conducted from 7 September 2023 to 5 October 2023 at selected public secondary schools in the Petaling district of Selangor, a state in Peninsular Malaysia.

### 2.2. Participants

In Malaysia, students aged 15 to 17 years enrol in Form 4 (equivalent to Grade 10 in many international systems). They were selected because they are still in their mid-adolescent stage, which is a critical developmental period marked by routine exposure to formal education systems and digital resources. This cohort was not involved in preparation for national public examinations, thereby mitigating potential confounding from stress levels and Internet usage patterns.

### 2.3. Procedure

A three-stage sampling procedure was employed: (i) simple random sampling to select nine schools out of 62 schools in the Petaling district (administrative division); (ii) proportionate sampling to determine the number of students from the selected schools; (iii) total sampling of Form 4 students from each class. For proportionate sampling, the OpenEpi^®^ software, Version 3.01 was used to calculate the sample size with Sullivan et al. (2009) formula. A minimum sample size of 1128 was determined based on an estimated population size (*N* = 17,565), design effect (DEFF = 3), anticipated proportion (*p* = 50%), and a desired absolute precision (d) at a 95% confidence level. After factoring in the potential dropout rate of 10%, this study targeted a total of 1241 students for the survey. The subset for mediation analysis was selected using proportionate random sampling to preserve the representativeness of the original sample across the participating schools. Approximately 44 respondents were randomly selected from each of five schools and 45 respondents from each of four schools, yielding a total sample of 400 participants for the measurement and structural model analyses.

The obtained sample size substantially exceeded the recommended minimum requirements for PLS-SEM and mediation analysis and was considered adequate to achieve stable parameter estimates and sufficient statistical power (Ajayi & Adebayo, 2021; Kline, 2017; Memon et al., 2020). PLS-SEM was employed because its prediction-oriented objective aligns with our research goals, and it effectively accommodates hierarchical component models (HCMs) through a two-stage approach; this allowed for the seamless estimation of higher-order constructs, the preservation of multidimensionality, and a significant reduction in model complexity while ensuring the total avoidance of indicator reuse bias.

Although covariance-based SEM is commonly used for theory confirmation, the present study primarily sought to estimate and predict relationships among higher-order latent constructs following measurement model validation. Therefore, PLS-SEM was considered the more appropriate analytical approach for this study’s objectives.

### 2.4. Measures

Formal permission was obtained for the adoption and translation of the Compulsive Internet Use Scale (CIUS), the Brief Scale of Psychological Well-Being for Adolescents (BSPWB-A), and the parent attachment domain of the Inventory of Parent and Peer Attachment (IPPA). The translation process from English to Malay was undertaken by bilingual experts using a rigorous forward–backward translation methodology in accordance with the recommended procedural guidelines (Ling et al., 2019). The Malay-adapted versions employed in this study were derived from previously validated psychometric work conducted among Malaysian adolescents. The construct reliability and validity of these versions were re-evaluated in the present sample.

Sociodemographic characteristics: Sociodemographic parameters in this study encompassed age, gender, weight, height, ethnicity, parental education, parental employment status, household size, and number of siblings.

Internet Usage Patterns: The assessment examined respondents’ digital device ownership, daily usage duration, peak access periods, and engagement across diverse online activities (e.g., social media, streaming, gaming, and educational platforms).

CIUS: The original CIUS consisted of 14 items, distributed across five distinct subdomains, namely loss of control (Items 1, 2, 5, and 9), preoccupation (Items 4, 6, and 7), withdrawal symptoms (Item 14), coping or mood modification (Items 12 and 13), and conflict (Items 3, 8, 10, and 11). Items were rated on a five-point Likert scale (0 = “Never” to 4 = “Very Often”), thereby yielding a total score between 0 and 56 (Meerkerk et al., 2008, p. 22; Pérez-Sáenz et al., 2023, p. 140). The original instrument demonstrated robust psychometric properties, as evidenced by Cronbach’s alpha (*α*) of 0.89 (Meerkerk et al., 2008). The Malay-adapted version used in this study comprised nine items, grouped into two subdomains: loss of control (six items) and preoccupation (three items).

BSPWB-A: Viejo et al. (2018) developed this instrument, which was an adaptation of Ryff’s original psychological well-being scale, comprising 20 items rated on a six-point Likert scale (1 = “Completely Disagree” to 6 = “Completely Agree”). The original instrument included four subdomains of self-acceptance, positive interpersonal relationships, autonomy, and life development (Viejo et al., 2018). The Malay-adapted version utilised in this study featured 16 items across four subdomains: self-acceptance (four items), autonomy (three items), continuous development (two items), and life experiences (two items).

IPPA: The parent attachment subscale of the revised IPPA was adapted by Gullone and Robinson (2005), comprising 28 items to assess adolescents’ perceptions of attachment to their parents across three subdomains: trust, communication, and alienation. Items were rated on a three-point Likert scale, with higher scores indicating greater agreement with each statement. The adapted Malay version used in this study comprised 14 items, organised into four subdomains: perceived care (five items), trust (four items), communication (three items), and alienation (three items).

### 2.5. Ethical Approval

Ethical approval for this study was obtained from the Research Ethics Committee. Additionally, permission to conduct the study was obtained from the management of each participating school. Informed consent was obtained from both parents or legal guardians and adolescent participants prior to data collection. All procedures were carried out in accordance with the Declaration of Helsinki, thereby upholding ethical research standards.

### 2.6. Data Collection Procedure

Parents and legal guardians were informed of the study through official communication distributed by the school management, which outlined the study’s purpose and procedures. Only students whose parents or legal guardians provided written consent and who themselves assented to participate in the study were included in the sample. Data collection was conducted using a paper-based questionnaire administered in a classroom setting. Prior to the distribution of the questionnaire, respondents received verbal instructions on how to complete it. Each session lasted approximately one hour and took place in a quiet, minimally disruptive environment, ensuring optimal data quality and participant focus.

### 2.7. Data Analysis

Data analysis was performed using IBM SPSS Statistics (Version 30) and SmartPLS (Version 4). A comprehensive manual review of the raw dataset was conducted to minimise data entry errors, detect missing-response patterns, and address ambiguous or extreme-value distributions, thereby enhancing data integrity. Outliers were identified using z-scores, and normality was assessed through skewness and kurtosis. Prior to the statistical analysis, all variables were systematically coded, and reverse scoring was applied to all negatively worded items to ensure consistency in scale direction. Missing data management was carried out using a structured methodological approach. As for cases with more than 10% missing values across key variables, listwise deletion was applied to prevent statistical bias. Datasets with missing values for essential sociodemographic characteristics, such as age, gender, ethnicity, parental employment status, number of siblings, and household size, were excluded. For continuous variables with less than 10% missing data, mean imputation was used. Descriptive analysis was conducted on the full dataset of 1598 respondents. For the mediation analysis, partial least squares–structural equation modelling (PLS–SEM) was performed on a randomly selected subset of 400 respondents.

In line with J. F. Hair et al. (2021) guidelines, SmartPLS (Version 4) was utilised to assess both the measurement and structural models. PLS–SEM was employed because the study involved multidimensional constructs modelled as hierarchical component models (HCMs) using the two-stage approach and aimed to explain and predict the relationships among PIU, parent–adolescent relationship quality, and psychological well-being. In the first stage, lower-order components and their indicators were estimated to establish reliability and validity, and latent variable scores were extracted. In particular, PIU comprised loss of control and preoccupation; PWB comprised self-acceptance, autonomy, continuous development, and life experiences; and PAR comprised perceived care, trust, communication, and alienation. In the second stage, these latent variable scores were used as indicators of their respective higher-order constructs in the structural model. The assessment of the measurement model included indicator reliability, internal consistency reliability, convergent validity, and discriminant validity. As for the structural model, path coefficients, coefficients of determination (*R*^2^), association sizes (*f*^2^), and predictive relevance (*Q*^2^) were estimated. Bootstrapping with 5000 resamples was employed to test the proposed hypotheses, with statistical significance set at *p* < 0.05 (Sarstedt et al., 2020).

## 3. Results

The total number of questionnaires collected from students in selected classes was 1921. After excluding 323 responses that were incomplete or contained data errors, the final analysis comprised 1598 valid responses.

### 3.1. Sociodemographic Characteristics and Internet Use

Table 1 presents the sociodemographic characteristics of the survey respondents. The sample consisted predominantly of 16-year-old students (M = 16.0, SD = 0.42), with a nearly equal gender distribution and the majority identifying as Malay. Most parents had completed at least secondary education, and the majority were employed. Over two-thirds of the respondents lived in households with four to six members and had three to five siblings.

As shown in Table 2, mobile phones were the most commonly owned device, and over half of the respondents used multiple digital tools. Daily Internet use was substantial: 78% of respondents reported spending five hours or more online per day. Peak usage occurred in the late afternoon and evening, with a high proportion (70.5%) reporting digital content creation. Such trends indicated extensive integration of Internet access into adolescents’ daily routines across academic, social, and recreational domains, thereby highlighting its developmental relevance.

### 3.2. Validation of Psychometric Properties of Study Instruments

#### 3.2.1. Adaptation of the Validated Malay Version of the CIUS

The original one-factor model entailed five correlated error terms. As demonstrated by parallel analysis and EFA, the two-domain model led to the reduction of the instrument to a nine-item CIUS. The indicators of Subdomain 1 measured “LC”, and those in Subdomain 2 measured “PO”. Outer loadings ranged from 0.562 to 0.762. Those with values under 0.7 were not deleted, as these items did not contribute to an increase in AVE. Referring to J. Hair et al. (2023), loadings of 0.4 or higher are deemed acceptable. The goodness-of-fit values indicated acceptable model fit. The original one-factor model with five correlated error terms was compared with the two-factor model (see Table 3), with the latter demonstrating adequate fit indices for the representative sample (Okafor-Emeagha et al., 2025).

#### 3.2.2. Adaptation of the Validated Malay Version of the BSPWB-A

The original model contained 20 items and four subdomains (Gómez-López et al., 2019). In this study, the EFA with parallel analysis generated four factors with 16 items. Some indicators with outer loadings of 0.323 (Item 10), 0.414 (Item 4), 0.5028 (Item 14), 0.478 (Item 13), and 0.512 (Item 18) were deleted because they increased *α* and AVE. The four subdomains in this study featured 11 items with adequate fit indices. The original four-factor model with 20 items was compared with the four-factor model with 11 items, which demonstrated fairly suitable model fit indices. Regardless, RMSEA remained above 0.08 (see Table 3).

#### 3.2.3. Adaptation of the Validated Malay Version of the Parent Attachment Domain

The original IPPA paradigm consisted of a 28-item, three-subdomain model of the parent attachment domain and a 25-item, three-subdomain model of the peer attachment domain (Gullone & Robinson, 2005). The factorial structure generated by parallel analysis and EFA, specifically a four-subdomain model with 18 items, was intended for the parent attachment domain. Based on the EFA, the subdomains of the parent attachment domain were renamed as “perceived care”, “trust”, “communication”, and “alienation”, in reference to Gullone and Robinson’s (2005) work. Responses on a three-point Likert scale for the negatively worded items in the alienation subdomain, such as “*I feel embarrassed when expressing my concerns to my parents*” and “*I can’t rely on my parents*”, were reverse-coded. The parent attachment domain demonstrated poor goodness-of-fit indices and low standardised outer weight loadings for some items. Those with lower loadings, such as Item 1 (0.486), Item 3 (0.476), Item 13 (0.464), and Item 25 (0.494), were deleted to improve goodness of fit, reliability, and validity. This resulted in four subdomains with 14 items (see Table 3).

### 3.3. Descriptive Statistics

Questionnaires with the following attributes were excluded: (1) incomplete data on sociodemographic characteristics (e.g., age, gender, and ethnicity); (2) errors in the response selection (not adhering to the instruction to choose a single option); (3) more than 10% of questions skipped. The response rate of 82.2% was deemed acceptable for survey-based research and indicated robust participation. Prior to hypothesis testing, a normality test, which included visual inspection, descriptive statistics (central tendency, skewness, and kurtosis), and formal statistical analysis, was conducted to assess whether the dataset was normally distributed.

When a large sample size is involved, skewness values between −2 and +2 and kurtosis values between −7 and +7 are considered acceptable for normality (J. F. Hair, 2009). Table 4 presents the obtained skewness and kurtosis values for all the variables in this study. Based on these metrics and visual inspection, data were found to align with the assumptions of normality. In addition, variance inflation factor values ranged from 1.000 to 1.184, indicating that the variables were not completely uncorrelated and that standard errors were not inflated. The descriptive statistics presented were based on the full sample (*N* = 1598), whereas the subsequent analyses of measurement and structural models were conducted using a randomly selected subset of 400 respondents. Similar structural relationships were observed when analyses were repeated using the full sample (*N* = 1598), supporting the stability of the randomly selected subset.

#### 3.3.1. Problematic Internet Use

The overall mean score for problematic Internet use, rated on a 5-point Likert scale, was M = 2.64, SD = 0.71, reflecting a moderate level of problematic Internet use in this sample. Further subdomain analysis (Table 4) showed a higher score for Loss of Control (M = 2.79, SD = 1.09) than for Preoccupation (M = 2.47, SD = 0.79), suggesting greater difficulties with Loss of Control than with Preoccupation.

#### 3.3.2. Psychological Well-Being

The overall mean score for psychological well-being, rated on a 6-point Likert scale, was M = 4.03, SD = 0.66, indicating moderate levels of psychological well-being among the adolescents. Among the constituent subdomains (Table 4), Continuous Development (CD) had the highest mean (M = 4.50, SD = 1.05), indicating a moderate-to-high level of engagement with learning and self-improvement initiatives. Life Experiences (LE) had a mean of M = 4.01 and an SD of 0.95, indicating a moderate inclination towards engaging in new and meaningful experiences. Self-Acceptance (SA) scores also indicated moderate levels of self-regard and resilience. Conversely, the Autonomy (AT) subdomain exhibited the lowest mean (M = 3.83, SD = 1.08), indicating comparatively lower levels of independent decision-making capacity among the participant cohort.

#### 3.3.3. Parent–Adolescent Relationship

The derived overall mean score for the parent–adolescent relationship construct, assessed using a 3-point Likert scale with the Malay version of the IPPA, was M = 2.18, SD = 0.39, indicating a moderate level of attachment between adolescents and their parents. Among the sub-domains (Table 4), Perceived Care (PC) had the highest mean (M = 2.50, SD = 0.65), indicating moderate-to-high perceptions of parental care. Trust (TR) and Communication (COM) exhibited comparable mean scores, indicating moderate levels of parental trust and communication, respectively. Notably, the Alienation (AL) subdomain registered the lowest score (M = 1.93, SD = 0.74), thereby indicating that adolescents predominantly reported low levels of emotional distance or disconnection from their parents.

### 3.4. Structural Model Results and Hypothesis Testing

As shown in Table 5, the two-factor model of the CIUS–Malay version demonstrated adequate construct reliability, with an overall *α* of 0.797. Its composite reliability (rho_c) and convergent validity were also found to be adequate. Likewise, the constructs in the four-factorial model of the BSPWB-A–Malay version demonstrated good *α* values and an overall internal consistency of 0.781. Results also demonstrated adequate convergent validity. The parent attachment domain in the Malay version demonstrated acceptable *α* values, an overall internal consistency of 0.709, and adequate convergent validity.

The model accounted for 25.7% of total variance in PWB (*R*^2^ = 0.259) and 4.8% of total variance in the parent–adolescent relationship (*R*^2^ = 0.048). The analysis of association size was conducted in accordance with Cohen’s guidelines. Results revealed a modest association between PIU and both the parent–adolescent relationship (*f*^2^ = 0.051) and PWB (*f*^2^ = 0.058). The parent–adolescent relationship demonstrated a moderate association with PWB (*f*^2^ = 0.149). Meanwhile, the model’s predictive capability was assessed using Stone–Geisser’s *Q*^2^ test. Notably, *Q*^2^ values for both endogenous constructs exceeded 0, indicating acceptable predictive relevance. As shown in Table 5, parent–adolescent relationship recorded *Q*^2^ of 0.047 (small predictive relevance), while PWB recorded *Q*^2^ of 0.072 (medium predictive relevance). Following the item reduction procedure, LE was not retained as a single-indicator construct because it did not demonstrate reliability indices (e.g., *α*). Its composite reliability was not estimated conventionally for single-item constructs. Therefore, it was deleted.

#### 3.4.1. Path Coefficients and Significance Testing

**Figure 2:**
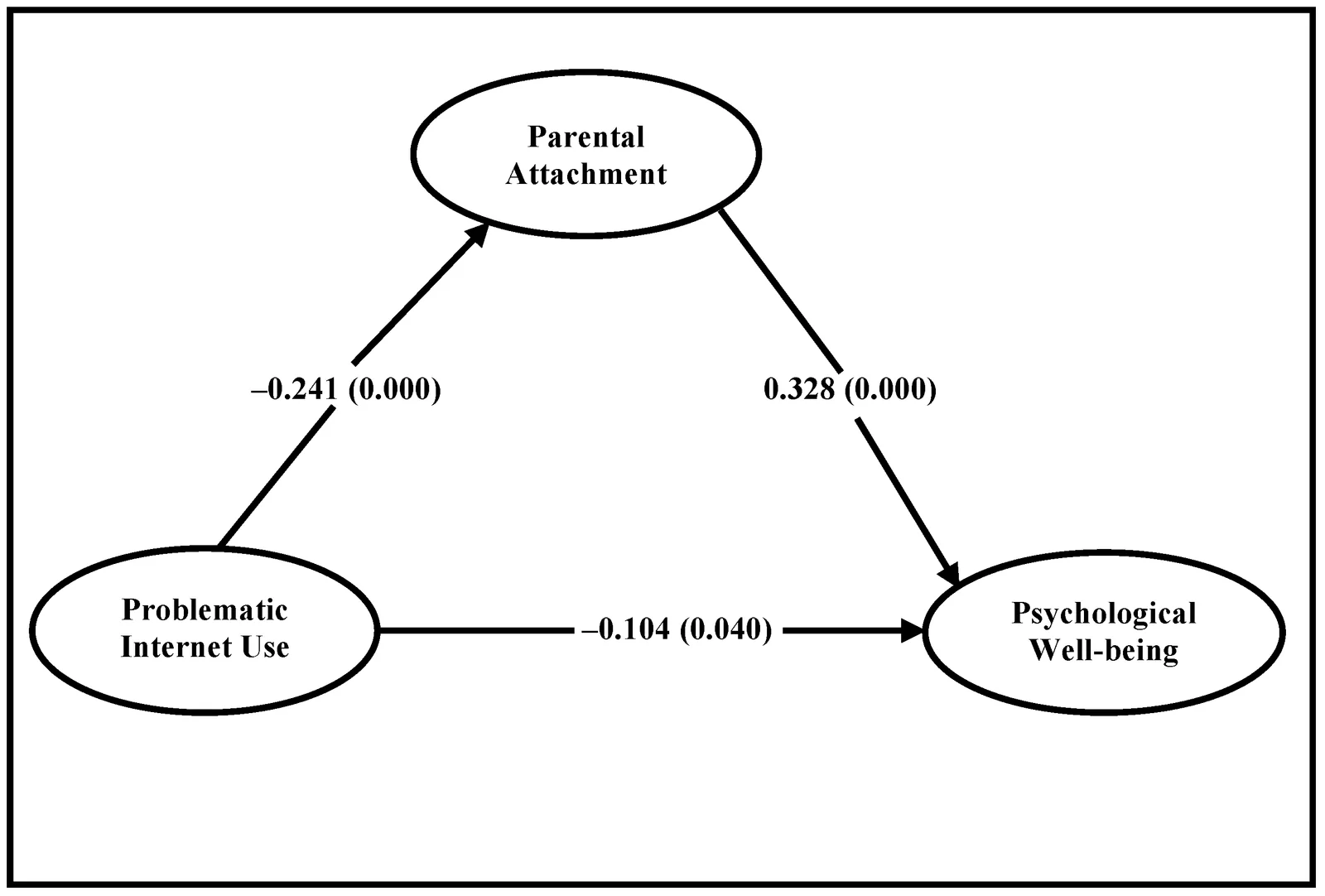
Mediation Model with Constructs (Subset Data *N* = 400).

PLS-SEM was conducted using SmartPLS to assess the structural relationships of PIU, PAR, and PWB. From Figure 2, PIU demonstrated a statistically significant and negative association with both PWB (*β* = −0.104, *p* = 0.040) and PAR (*β* = −0.241, *p* < 0.001). PAR also demonstrated a statistically significant positive association with PWB (*β* = 0.328, *p* < 0.001). These results consequently supported H1 and H2, which indicated that elevated PIU levels were associated with diminished PWB and attenuated PAR. Furthermore, positive PAR quality was associated with optimal PWB (H3).

**Figure 3:**
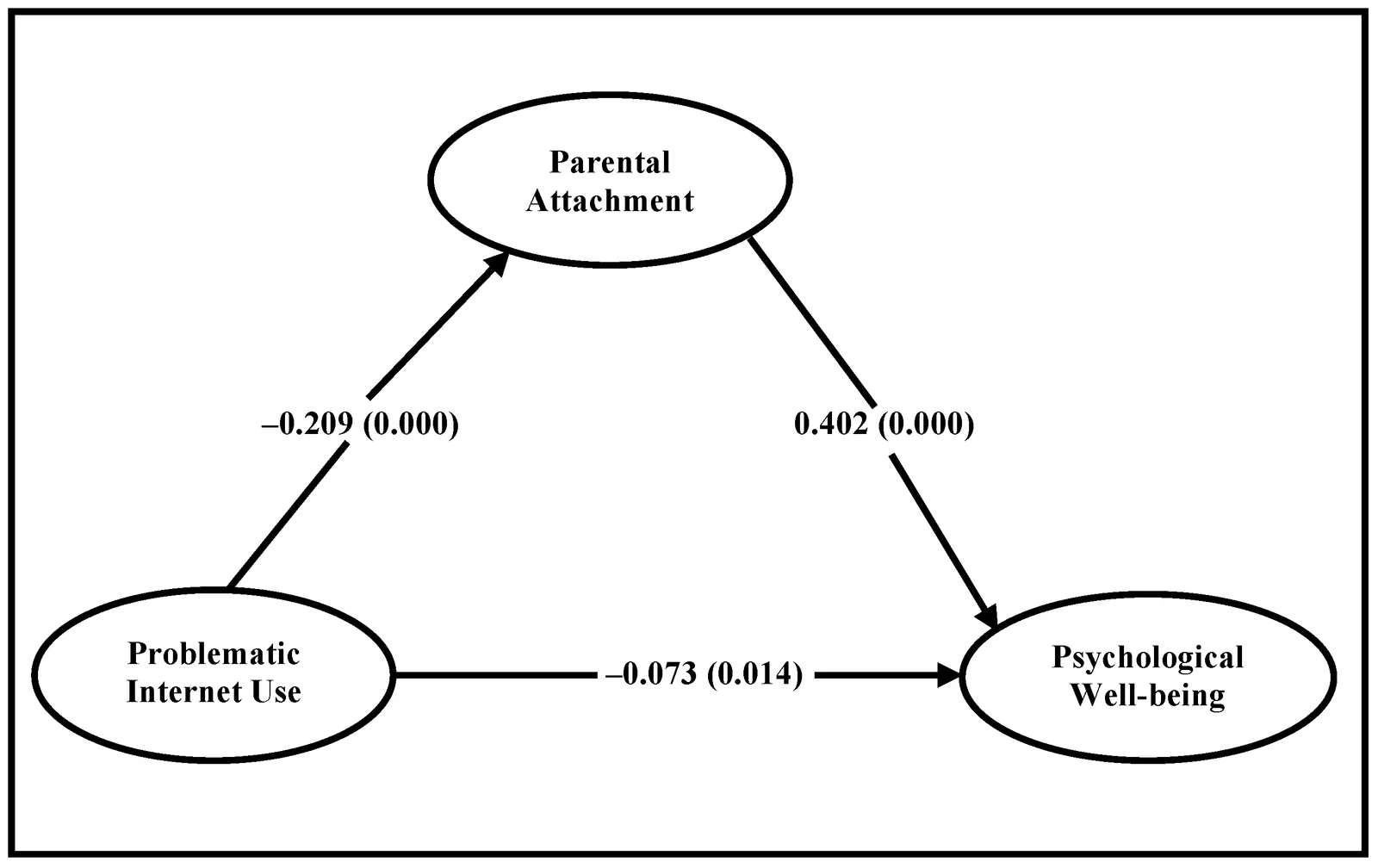
Mediation Model with Constructs (Full Data *N* = 1598).

Additional analyses were conducted to compare the random subset data (400) with the full sample (1598) (Figure 3). The comparison between the subset data and the full sample still yielded stable and comparable results. PIU is significantly and negatively associated with PWB and PAR, and findings from both samples demonstrate a partial mediating effect. Detailed comparisons of Figure 2 and Figure 3 can be found in the Supplementary Materials Section.

#### 3.4.2. Mediation Analysis

Bootstrapping with 5000 resamples was employed to evaluate indirect associations. Results demonstrated that PAR significantly mediated the relationship between PIU and PWB (*β* = −0.079, *p* < 0.001), thereby supporting H4. The observed mediation in this relationship was partial, given that the direct relationship remained statistically significant (*β* = −0.104, *p* = 0.040), and the total effect was notably stronger (*β* = −0.183, *p* < 0.001), as presented in Table 6. This suggested that, while PAR might mitigate the negative relationships involving PIU, its detrimental influence on adolescents’ well-being persisted.

## 4. Discussion

This study examined the structural relationships among PIU, PWB, and PAR, and assessed PAR’s mediating role between PIU and PWB.

### 4.1. Adolescent Problematic Internet Use

Results revealed that adolescents in this study exhibited moderate levels of PIU, a condition consistent with trends observed in regional studies (Afolabi et al., 2022). In this context, technology use emerged as a key environmental stimulus, thereby influencing cognitive, emotional, and behavioural development. Elevated scores in LC were consistent with prior studies. This reflected neurodevelopmental immaturity of the prefrontal cortex in adolescence, with consequent impairments in their abilities to regulate impulses, make decisions, and exercise behavioural restraints (Dalamaria et al., 2020; Díaz-López et al., 2021). Conversely, PO’s lower scores indicated evident early-stage signs of dependency among the adolescents who have not yet reached complete behavioural immersion. This suggested a transitional phase in which habitual usage patterns are forming, even if they have not yet displaced essential activities. Such a behavioural profile signals a critical window for early intervention to prevent progression towards severe addiction. Overall, the moderate level of PIU observed in this study underscored the need for preventive strategies and digital literacy interventions targeted at early adolescence, focusing on impulse control, time management, and awareness of healthy Internet use amid increasing digital integration across academic and social domains.

### 4.2. Psychological Well-Being of Adolescents

Overall, moderate levels of autonomy, continuous development, and life experiences were observed among adolescents in this study. AT, reflecting the ability to make independent decisions and to resist undue social pressure, was consistent with developmental expectations for mid-adolescence, when independence emerged yet remained associated with ongoing parental or institutional guidance. However, in testing the structural model, AT demonstrated no direct relationship with PWB in both the subset data and the full sample. This may suggest that in this specific target population, adolescents’ general psychological well-being does not structurally drive or alter their level of independence (autonomy).

Meanwhile, CD scores indicated willingness to engage in personal growth and learning, suggesting adaptive coping and resilience. LE scores reflected openness to new experiences, suggesting healthy psychological adjustment. While these findings were encouraging, they contradicted global reports of declining youth mental health (Varadkar & Gadgil, 2024). The moderate scores across these subdomains highlighted the need for targeted interventions to strengthen autonomy and emotional coping strategies during this formative period, thereby promoting optimal psychosocial development.

### 4.3. Parent–Adolescent Relationships

Respondents reported low-to-moderate parental relationship quality, aligning with existing empirical evidence that emotional connectedness, parental supervision, and trust often decline during adolescence (Sugimura et al., 2023; Waters et al., 2019). This developmental period is characterised by an increase in behavioural and emotional autonomy, which may contribute to diminished closeness and communication with parents (Waters et al., 2019). In the absence of intentional efforts to maintain open communication, these developmental shifts can lead to a perceived reduction in parental support. The Bioecological Model of Human Development emphasised the significance of proximal processes in shaping psychosocial outcomes, specifically reciprocal interactions between parents and adolescents (Gard et al., 2022). Diminished communication and trust may inhibit disclosure and help-seeking for personal concerns, limiting opportunities for early detection of risky behaviours (e.g., PIU). Consequently, targeted interventions that equip parents to consider adolescents’ perspectives, recognise emotional and behavioural cues of psychological distress, and adopt supportive communication styles that promote discussion are recommended to strengthen relational bonds and foster adolescents’ resilience.

### 4.4. Problematic Internet Use and Psychological Well-Being

This study’s results on the significant negative relationship between PIU and PWB (H1) aligned with prior studies linking excessive Internet use with adverse mental health outcomes, including depression, anxiety, and emotional dysregulation (Dermani & Perdikaris, 2022; Soriano-Molina et al., 2025). A bidirectional relationship may be plausible; specifically, psychological vulnerabilities may predispose adolescents to maladaptive online coping mechanisms, subsequently exacerbating existing mental health challenges (Trumello et al., 2021). From a developmental standpoint, limited emotional regulation skills and restricted offline support networks may lead adolescents to rely on digital platforms for social validation and distraction. Within Bronfenbrenner’s Bioecological Model of Human Development, the Internet functions as a proximal environmental stimulus that can either support or hinder developmental trajectories, contingent on the Internet usage patterns. This study’s findings underscore the essential importance of early detection and preventive programmes that combine digital literacy with emotional resilience training to facilitate optimal psychosocial development in adolescents.

### 4.5. Problematic Internet Use and Parent–Adolescent Relationships

The significant negative relationship observed between PIU and PAR (H2) in this study supported previous findings that weakened parental attachment and less supportive family environments are associated with greater susceptibility to excessive Internet use among adolescents (Agung et al., 2018; Ballarotto et al., 2018). Conversely, supportive and emotionally responsive home environments exert a protective influence over maladaptive Internet behaviours. Adolescents who perceive greater familial alienation or diminished parental warmth may be more likely to seek online environments for compensatory psychosocial fulfilment and relational engagement (Hwang & Toma, 2021; Sarfika et al., 2023). Such compensatory online engagement may gradually become habitual and further strain parent–adolescent interactions. These reciprocal patterns of increasing Internet engagement and weakening interpersonal relationships have been reported across both Western and Asian contexts (Yankouskaya et al., 2025). Interventions should adopt a multifaceted approach that addresses not only adolescents’ digital behaviours but also the quality of family interactions and communication.

Notably, PIU explained only 4.8% of the total variance in PAR, indicating PIU’s relatively modest explanatory contribution despite its statistically significant relationship with PAR. From a bioecological perspective, PAR is shaped by multiple interacting influences within adolescents’ immediate environments. Therefore, a substantial proportion of the variance in relationship quality in this study is likely attributable to other microsystem factors, including parenting practices, attachment experiences, peer interactions, and academic stressors. This suggested the potential influence of PIU on relationship difficulties. However, it may be unlikely to be the primary determinant of family relationship quality. Future research should examine PIU alongside these broader family and contextual factors to develop a more comprehensive understanding of adolescents’ relational well-being.

### 4.6. Parent–Adolescent Relationships and Psychological Well-Being

The significant positive relationship between PAR and PWB (H3) in this study supports previous findings that positive parent–adolescent relationships promote optimal wellbeing (Guy-Evans, 2020). In Phase 3 of the Bioecological Model, Bronfenbrenner introduced the concept of proximal processes. These are regular, face-to-face forms of interaction in the immediate environment. Parent–adolescent interactions, such as daily conversations, shared meals, conflict resolution, and emotional support, are the ultimate proximal processes. When these processes are positive, high-quality, and consistent, they help actualise an adolescent’s potential for resilience, emotional regulation, and self-esteem. Thus, optimal well-being is the direct psychological output of these highly functional, everyday interactions (Li et al., 2023; Zhu & Shek, 2021).

### 4.7. Mediating Role of Parent–Adolescent Relationships

The results of mediation analysis in this study demonstrated the partial mediating effect of PAR on the established relationship between PIU and PWB (H4), which aligned with extant empirical evidence indicating that parental warmth, consistent monitoring, and supportive communication can reduce adolescents’ vulnerability to psychosocial risks associated with PIU (De Simone et al., 2025). From a bioecological systems perspective, PAR constitutes a critical microsystem factor. Therefore, proactive parental mediation strategies, encompassing modelling healthy technology use, establishing dedicated tech-free periods, and actively promoting offline activities, can foster resilience and concomitantly reduce reliance on virtual environments for emotional fulfilment, thereby supporting adolescents’ developmental trajectories.

### 4.8. Implications and Recommendations

The current study’s findings suggested the need to adopt a multilevel approach to strengthen PAR as part of the strategic interventions for adolescents’ PIU. At the individual level, interventions should promote digital self-regulation skills, particularly strategies that address adolescents’ LC and PO with Internet use. At the family level, interventions should strengthen communication, trust, and emotional connectedness between parents and adolescents, as these relational factors may protect against poorer psychological well-being. From a policy perspective, school-based digital literacy programmes should integrate healthy technology use with parent–adolescent communication and relationship-building components to promote adolescents’ digital well-being.

### 4.9. Limitations and Future Research

Several limitations in this study warrant consideration. Firstly, this study’s cross-sectional design precluded causal inferences. Secondly, the reliance on self-report measures introduced potential biases, including social desirability associations. Thirdly, the focus on a specific age group (Form 4 students in Malaysia) limited the generalisability of this study’s findings to other adolescent populations. Further longitudinal investigation is necessary to examine these relationships over time. The weak structural path between PWB and AT also requires further investigation to ascertain whether a significant relationship exists between them and to examine whether the indicators used to measure AT accurately capture adolescents’ interpretations of independence. While this study focused on the mediating role of PAR, there is a need to explore additional mediating and moderating variables, including peer relationships and school connectedness, thereby enhancing the understanding of complex developmental trajectories.

### 4.10. Strengths of the Study

Despite the identified limitations, this study demonstrated several notable strengths. A notable strength of this study resided in its comprehensive, theory-driven design, anchored in Bronfenbrenner’s Bioecological Model of Human Development. This theoretical framework enabled this study to examine how individual, relational, and contextual factors interact to influence adolescents’ PIU and PWB. By incorporating both proximal (e.g., parental relationships) and contextual (e.g., school and digital environments) associations, this study provided a nuanced understanding of adolescents’ digital behaviour in the Malaysian context. Another key strength involved the use of total (universal) sampling and the integration of correlational and multivariate analyses (including mediation analysis), thereby strengthening the study’s methodological robustness. These analytical approaches allowed the investigation of both direct and indirect pathways, contributing to theoretical refinement and practical insights.

## 5. Conclusions

Grounded in Bronfenbrenner’s Bioecological Model of Human Development, this study identified PIU as a significant proximal process negatively affecting Malaysian adolescents’ PWB. PAR partially mediated this relationship, highlighting the protective role of the family microsystem. This study’s findings underscore the need for targeted interventions involving families, educators, and health professionals.

## Figures and Tables

**Table 1:** Sociodemographic Background of Adolescents (*N* = 1598).

Variables/Categories		Frequency (*n*)	Percentage (%)	Mean (SD)	Range
Age (years)				16 (0.42)	
15		213	13.3		
16		1309	81.9		
17		76	4.8		
Gender					
Male		778	48.7		
Female		820	51.3		
BMI					
Normal		1079	67.6		
Underweight		269	16.8		
Overweight		146	9.1		
Obese		104	6.5		
Ethnicity					
Malay		1178	73.7		
Chinese		196	12.3		
Indian		180	11.3		
Others		44	2.8		
Father’s educational level					
No formal education		35	2.2		
Primary school		36	2.3		
Secondary school		625	39.1		
College/university		902	56.4		
Mother’s educational level					
No formal education		33	2.1		
Primary school		40	2.5		
Secondary school		550	34.4		
College/university		975	61.0		
Employment status	Father				
	Employed	1487	93.1		
	Unemployed	111	6.9		
	Mother				
	Employed	1095	68.5		
	Unemployed	500	31.5		
No. of people in a household				2 (0.53)	2
1–3		134	8.4		
4–6		1128	70.6		
7 or more		336	21.0		
Number of siblings					
0		72	4.5		
1–2		487	30.5		
3–5		901	56.4		
6 or more		138	8.6		

**Table 2:** Internet Usage Patterns among Adolescents (*N* = 1598).

Variables/Categories	Frequency (*n*)	Percentage (%)	Mean (SD)
Number of Digital Devices			3.21 (1.96)
Mobile phone	690	43.2	
Tablet	14	0.9	
Laptop	20	1.3	
Desktop	12	0.8	
More than one device	861	53.9	
None	1	0.1	
Hours Spent Online			2.03 (0.69)
Less than 5 h	352	22.0	
5–12 h	846	53.0	
12 h or more	400	25.0	
Popular Time Spent Online			3.14 (1.15)
6 a.m. to 12 p.m.	79	4.9	
12 p.m. to 4 p.m.	398	24.9	
4 p.m. to 9 p.m.	587	36.7	
10 p.m. to Midnight	365	22.8	
Midnight to 6 a.m.	88	5.5	
Different periods of the day	81	5.1	
Hours of Chatting Online			2.36 (0.81)
None	58	3.6	
1–3	1118	70.0	
4–6	284	17.8	
7–8	63	3.9	
9 h or more	75	4.7	
Hours on Social Media			2.95 (0.98)
None	38	2.4	
1–3	551	34.5	
4–6	612	38.3	
7–8	253	15.8	
9 h or more	144	9.0	
Hours of Content Creation			1.94 (0.63)
None	308	19.3	
1–3	1127	70.5	
4–6	127	7.9	
7–8	25	1.6	
9 h or more	11	0.7	
Hours of Reading Online			2.30 (0.79)
None	155	9.7	
1–3	954	59.7	
4–6	368	23.0	
7–8	89	5.6	
9 h or more	32	2.0	
Hours on Homework Online			2.33 (0.68)
None	74	4.6	
1–3	1032	64.6	
4–6	398	24.9	
7–8	81	5.1	
9 h or more	13	0.8	
Hours of Online Gaming			2.61 (0.99)
None	103	6.4	
1–3	798	49.9	
4–6	420	26.3	
7–8	169	10.6	
9 h or more	108	6.8	
Other Online Activities			2.32 (0.93)
None	216	13.5	
1–3	891	55.8	
4–6	327	20.5	
7–8	96	6.0	
9 h or more	68	4.3	

**Table 3:** Comparison of Model Fit before and after Modification.

Index	*Χ*^2^/*df*	RMSEA	CFI	SRMR	TLI
Best value	<3	<0.08	>0.90	<0.08	≥0.90
CIUS					
One-factor model (Original)	3.762	0.114	0.861	0.070	0.815
Two-factor model (Adopted)	2.266	0.077	0.942	0.048	0.920
BSPWB-A					
Four-factor model (Original)	4.626	0.131	0.655	0.145	0.600
Four-factor model (Adopted)	2.894	0.095	0.929	0.078	0.897
Parent Attachment Domain					
Three-factor model (Original)	3.014	0.098	0.577	0.108	0.536
Four-factor model (Adopted)	2.052	0.071	0.903	0.078	0.875

**Table 4:** Skewness and Kurtosis Values for Normality Assessment, Descriptive Statistics (*N* = 1598).

Variable	Mean (SD)	95% CI	Median	Skewness (SE)		Kurtosis (SE)
CIUS LC	16.52 (4.68)	(16.29, 16.75)	16.00	0.277 (0.061)		−0.104 (0.122)
CIUS PO	9.88 (3.17)	(9.73, 10.04)	10.00	0.377 (0.061)		−0.200 (0.122)
BSPWBA SA	15.79 (3.96)	(15.60, 15.99)	16.00	−0.283 (0.061)		0.476 (0.122)
BSPWBA AT	8.02 (1.91)	(11.32, 11.64)	12.00	−0.168 (0.061)		−0.211 (0.122)
BSPWBA CD	11.47 (3.25)	(8.89, 9.10)	9.00	−0.633 (0.061)		0.514 (0.122)
BSPWBA LE	9.00 (2.11)	(7.93, 8.11)	8.00	−0.424 (0.061)		0.880 (0.122)
PAR-PC	12.43 (2.12)	(12.32, 12.53)	13.00	−0.667 (0.061)		−0.251 (0.122)
PAR-TR	6.19 1.70)	(6.11, 6.28)	6.00	−0.110 (0.061)		−0.766 (0.122)
PAR-COM	5.79 (1.54)	(5.72, 5.88)	6.00	0.033 (0.061)		−0.651 (0.122)
PAR-AL	8.09 (2.24)	(7.99, 8.21)	8.00	−0.027 (0.061)		−0.777 (0.122)
**Descriptive Statistics for Variables (*N* = 1598)**						
Domain/Item				Mean (SD)		Range
Overall Problematic Internet Use (5-point LS)				2.64	0.71	2.50–2.67
Loss of Control (LC)				2.79	1.09	2.56–3.17
LC1.	Difficulty in stopping internet use			3.01	1.05	
LC2.	Continuing internet use even after intending to stop			2.98	1.07	
LC3.	Lack of sleep due to internet use			2.67	1.17	
LC4.	Failure to reduce internet time			2.76	1.07	
LC5.	Prefer the internet to spending time with family			2.56	1.13	
Preoccupation (PO)				2.47	0.79	2.00–3.00
PO1.	Thinking about the internet even when offline			2.39	1.03	
PO2.	Looking forward to the next internet session			2.54	1.07	
PO3.	Feeling anxious and frustrated when there is no internet			2.29	1.13	
PO4.	Rushing to finish schoolwork to use the internet			2.66	1.12	
Overall Psychological Well-being (6-point LS)				4.03	0.66	4.00–4.50
Self-acceptance (SA)				3.95	0.99	3.50–4.50
SA1.	Happiness and satisfaction with one’s life			4.11	1.18	
SA2.	Feeling confident and positive about oneself			3.85	1.22	
SA3.	Liking most aspects of one’s personality			3.88	1.15	
SA4.	Feeling proud of oneself			3.96	1.17	
Autonomy (AT)				3.83	1.08	3.00–4.67
AT1.	Worried about other people’s judgment			3.95	1.35	
AT2.	Worried about other people’s opinions			3.96	1.33	
AT3.	Feel disappointed with life’s achievements			3.56	1.32	
Continuous Development (CD)				4.50	1.05	4.00–5.50
CD1.	Life is a continuous process of learning			4.67	1.19	
CD2.	Finding a change during difficulties			4.33	1.18	
Life Experiences (LE)				4.01	0.95	3.50–4.50
LE1.	Change opinions if friends or family disagree			3.72	1.23	
LE2.	New challenging experiences are important			4.29	1.13	
Overall Parent–Adolescent Relationship (3-point LS)				2.18	0.39	2.00–2.60
Perceived Care (PC)				2.53	0.61	2.20–2.80
PC1.	My parents are good.			2.71	0.52	
PC2.	My parents accept me as I am.			2.57	0.63	
PC3.	I believe my parents.			2.58	0.61	
PC4.	My parents understand me.			2.24	0.68	
Trust (TR)				2.03	0.72	1.80–2.40
TR1.	When I get angry about something, my parents try to understand.			1.98	0.71	
TR2.	My parents helped me understand myself better.			2.16	0.74	
TR3.	My parents support me in expressing my concerns.			2.11	0.72	
TR4.	I tell my parents about the problems that I’m facing.			1.86	0.72	
Communication (COM)				2.05	0.74	2.00–2.15
COM1.	My parents can sense when I’m upset about something.			2.08	0.74	
COM2.	When my parents know that I’m upset about something, they ask about it.			2.05	0.74	
COM3.	My parents listen to how I feel.			2.02	0.75	
Alienation (AL)				1.93	0.74	1.70–2.12
AL1.	I can’t rely on my parents to help me solve problems.			1.84	0.73	
AL2.	I feel embarrassed when I express my problems to my parents.			1.93	0.79	
AL3.	It doesn’t help when I show my feelings when I’m upset.			2.03	0.74	

**Table 5:** Structural Model Testing (*N* = 400 Random Subset).

Construct Reliability and Validity											
			Outer Loading				*α*	rho_c		Ave	
			Initial		Modified						
Problematic Internet Use											
Loss of control							0.796	0.868		0.620	
LC1			0.778		0.809						
LC2			0.763		0.829						
LC3			0.702		0.710						
LC4			0.710		0.716						
LC5			0.637		deleted						
Pre-occupation							0.713	0.840		0.637	
PO1			0.766		0.815						
PO2			0.795		0.810						
PO3			0.710		0.747						
PO4			0.622		deleted						
Psychological Well-being											
Self-acceptance							0.860	0.903		0.701	
SA1			0819		0.823						
SA2			0.877		0.880						
SA3			0.817		0.813						
SA4			0.843		0.840						
Autonomy							0.845	0.928		0.866	
AT1			0.921		0.949						
AT2			0.922		0.909						
AT3			0.512		deleted						
Life Experiences											
LE1			0.216		deleted		Redundant/deleted				
LE12			0.997		1.000						
Continuous Development							0.740	0.885		0.794	
CD1			0.897		0.894						
CD2			0.885		0.888						
Parent Attachment											
Perceived Care							0.750	0.840		0.568	
PC1			0.713		0.732						
PC2			0.705		0.722						
PC3			0.791		0.794						
PC4			0.764		0.784						
Trust							0.781	0.859		0.603	
TR1			0.751		0.791						
TR2			0.717		0.766						
TR3			0.765		0.790						
TR4			0.734		0.760						
Communication							0.701	0.832		0.623	
COM1			0.748		0.748						
COM2			0.817		0.817						
COM3			0.805		0.806						
Alienation							0.605	0.770		0.627	
AL1			0.466		deleted						
AL2			0.784		0.817						
AL3			0.743		0.765						
**Discriminant Validity (Fornell–Larcker)**											
	AL	AT	COM	CD		LC	PC	PO	SA		TR
AL	0.781										
AT	0.203	0.783									
COM	0.199	0.127	0.778								
CD	0.008	−0.058	0.054	0.872							
LC	−0.077	−0.199	−0.170	−0.048		0.796					
PC	0.062	0.186	0.517	0.205		−0.178	0.786				
PO	−0.070	−0.085	−0.126	−0.153		0.419	−0.183	0.760			
SA	0.140	0.281	0.224	0.357		−0.129	0.309	−0.105	0.805		
TR	0.201	0.175	0.725	0.130		−0.194	0.612	−0.156	0.324		0.763
Results of Coefficient of Determination (*R*^2^)											
Endogenous Latent Variable				*R* ^2^				Adjusted *R*^2^			
Parent–adolescent relationship				0.048				0.048			
Psychological well-being				0.259				0.257			
Results of Association Sizes *f*^2^ for the Variables											
Variables						f-square					
PIU → Parent–adolescent relationship						0.051					
PIU → Psychological well-being						0.058					
Parent Relationship → Psychological well-being						0.149					
Results of Predictive Relevance (*Q*^2^)											
Endogenous Latent Variable						*Q*^2^ predict					
Parent–adolescent relationship						0.047					
Psychological well-being						0.072					

*R*^2^: Coefficient of Determination; Adjusted *R*^2^: Adjusted Coefficient of Determination. *Q*^2^: predictive relevance. Cronbach’s alpha, Convergent Validity (Average Variance Extracted (Ave) and composite reliability rho_c). Discriminant validity: LC: Loss of Control; PO: Pre-occupation; SA: Self-acceptance; AT: Autonomy; CD: Continuous Development; PC: Perceived care; COM: Communication; TR: Trust; AL: Alienation.

**Table 6:** Hypothesis Testing Summary and Mediation Analysis (*N* = 400).

Hypothesis		Path	*β*	*p*	Results
H1: There is a significant association of problematic internet use with psychological well-being.		PIU→PWB	−0.104 *	0.040	Supported
H2: There is a significant association between adolescent problematic internet use and their relationship with their parents.		PIU→PAR	−0.241 **	0.001	Supported
H3: There is a significant association between parent–adolescent relationship and psychological well-being.		PAR→PWB	0.328 **	0.001	Supported
H4: The quality of parent–adolescent relationships mediates the relationship between problematic internet use and psychological well-being.		PIU→PAR→PWB	−0.079 **	0.001	Supported
Mediation Analysis—Direct, Indirect, and Total Effect					
Path	Mediator	Total effect	Direct effect	Indirect effect	Results
Subset Data (*N* = 400)					
PIU -> PWB	PAR	−0.183 ** (*p* < 0.001)	−0.104 * (*p* = 0.040)	−0.079 ** (*p* < 0.001)	Partial positive mediation
Full Data (*N* = 1598)					
PIU -> PWB	PAR	−0.157 ** (*p* < 0.001)	−0.073 * (*p* = 0.014)	−0.084 ** (*p* < 0.001)	Partial positive mediation

** *p* < 0.01; * *p* < 0.05; PIU: Problematic Internet Use; PWB: Psychological well-being; PAR: Parent–adolescent relationship.

## Data Availability

The data presented in this study are available on request from the corresponding author due to privacy and ethical restrictions.

## Supplementary Materials

The following supporting information can be downloaded at: https://www.mdpi.com/article/10.3390/bs16081341/s1.

## Abbreviations

AL	Alienation
AT	Autonomy
AVE	Average Variance Extracted
BSPWBA	Brief Scale of Psychological Well-being for Adolescents
CD	Continuous Development
CFA	Confirmatory Factor Analysis
CFI	Comparative Fit Index
CIUS	Compulsive Internet Use Scale
COM	Communication
EFA	Exploratory Factor Analysis
IPPA	Inventory of Parents and Peers Attachment
LC	Loss of Control
LE	Life Experiences
PC	Perceived Care
PAR	Parent Adolescent Relationship
PIU	Problematic Internet Use
PLS-SEM	Partial Least Squares Structural Equation Modelling
PO	Preoccupation
PWB	Psychological Well-being
RMSEA	Root Mean Square Error of Approximation
SA	Self-acceptance
SRMR	Standardised Root Mean Square Residual
TLI	Tucker–Lewis Index
TR	Trust
