# Supplementary material for: Beyond Screen Time: Parent–Adolescent Relationship as a Mediating Pathway Between Problematic Internet Use and Psychological Well-Being Among Malaysian Adolescents
Source: Behav Sci (Basel). 2026 Aug 4;16(8):1341. doi: 10.3390/bs16081341 (PMC13510131; doi:10.3390/bs16081341)
Supplement: Supplementary file 1 [file behavsci-16-01341-s001.zip › behavsci-4328430-supplementary.pdf]

STRUCTURAL MODEL WITH SECOND-ORDER AND LOWER-ORDER COMPONENTS

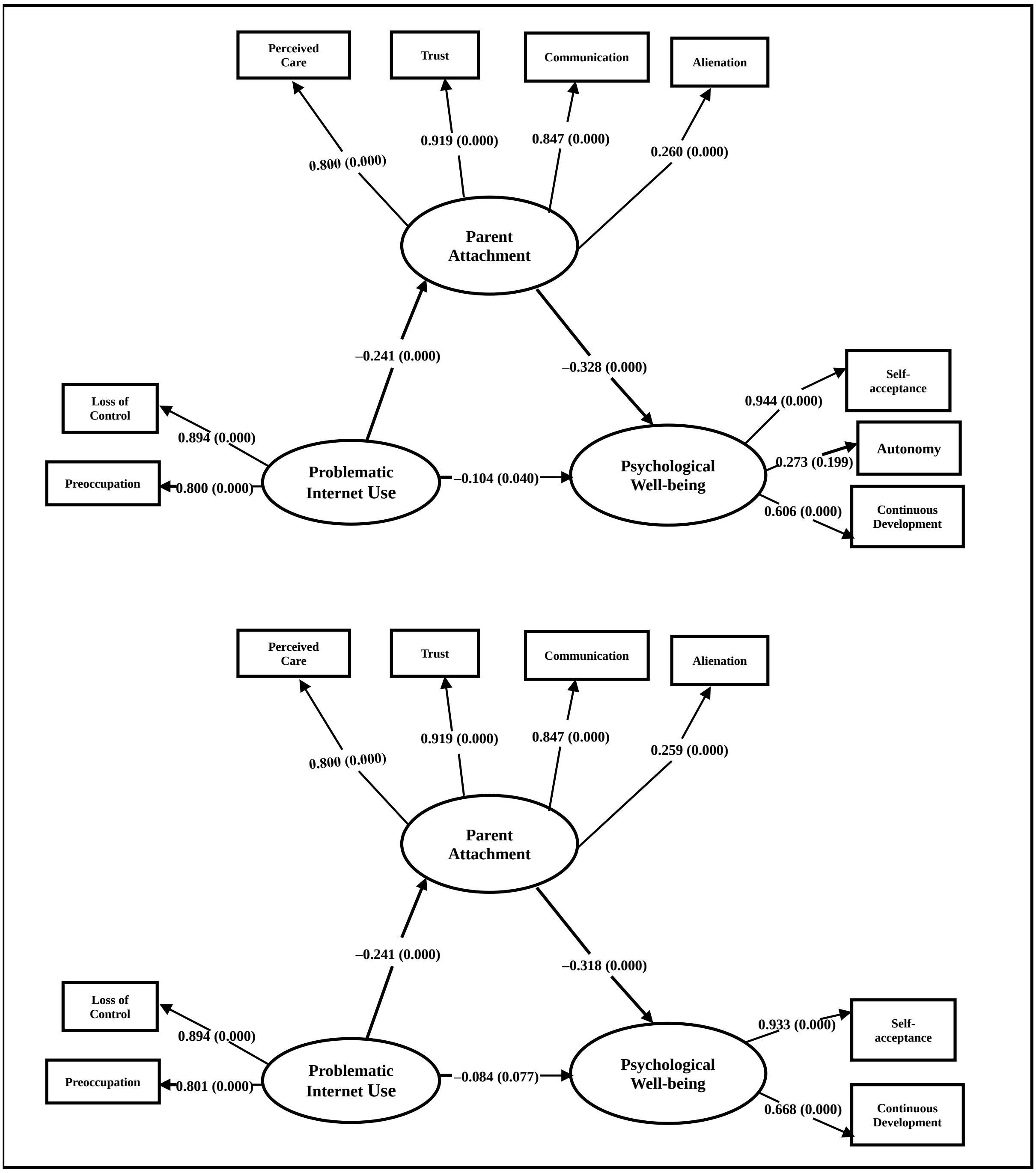

Figure S1. Mediation Model with Constructs (Subset Data N = 400).

From Figure S1, PIU demonstrated a statistically significant and negative association with both PWB (β = –0.104, p = 0.040) when AT was retained and PAR (β = –0.241, p < 0.001). However, when AT was removed in the subset data (N = 400), it impacted the p-value (p = 0.077).

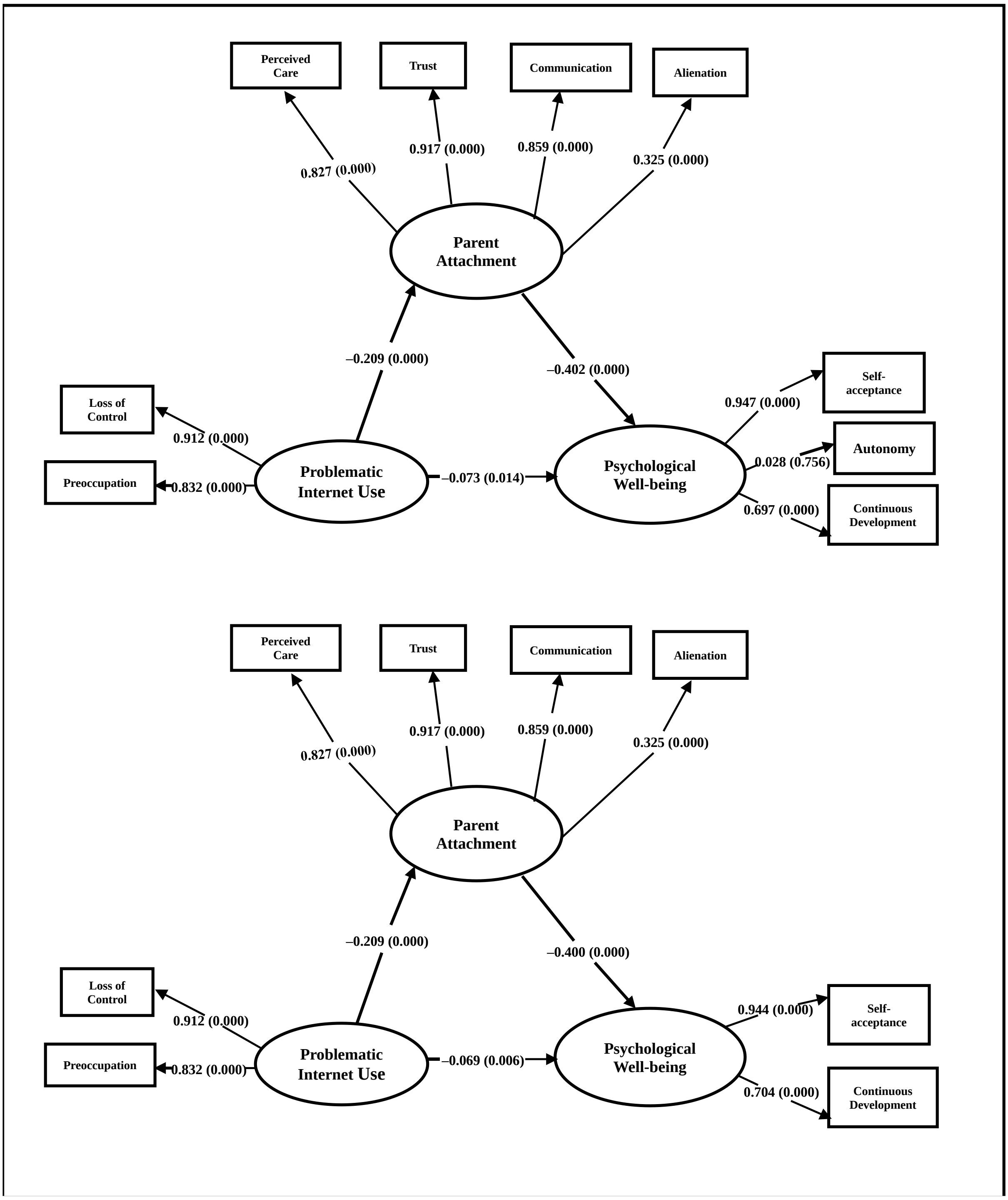

Figure S2. Mediation Model with Constructs (Full Data N = 1,598).

The comparison between the subset data and the full sample still yielded stable and comparable results, except for the lack of statistical significance between PWB and AT.

Table S1. Mediation Analysis – Direct, Indirect, and Total Effect.

Path	Mediator	Total effect	Direct effect	Indirect effect	Results
**Subset Data (*N* = 400)**					
PIU -> PWB	PAR	−0.183** (p<0.001)	−0.104* (p=0.040)	−0.079** (p<0.001)	Partial positive mediation
**Subset Data (*N* = 400, excluding AT)**					
PIU -> PWB	PAR	−0.161** (p<0.001)	−0.084 (p=0.077)	−0.077** (p<0.001)	Partial positive mediation
**Full Data (*N* = 1,598)**					
PIU -> PWB	PAR	−0.157** (p<0.001)	−0.073* (p=0.014)	−0.084** (p<0.001)	Partial positive mediation
**Full Data (*N* = 1,598, excluding AT)**					
PIU -> PWB	PAR	−0.153** (p<0.001)	−0.069* (p=0.006)	−0.084** (p<0.001)	Partial positive mediation

**p <0.01; *<0.05; PIU: Problematic Internet Use; PWB: Psychological well-being; PAR: Parent-adolescent relationship, AT: Autonomy.
